# Effects of dyadic patterns and proficiency pairing on Chinese EFL learners’ second language learning in collaborative writing

**DOI:** 10.3389/fpsyg.2022.1004924

**Published:** 2022-10-20

**Authors:** Ningning Li, Yingliang Liu

**Affiliations:** School of Foreign Languages, Wuhan University of Technology, Wuhan, China

**Keywords:** collaborative writing, second language learning, dyadic patterns, proficiency pairing, language-related episodes

## Abstract

Research on Collaborative Writing (CW) has proliferated over recent decades, but the role that pair dynamics plays in second language (L2) learning remains unclear. This study compared the effect of dyadic interactions and proficiency pairing in CW on L2 learning. Sixty-two Chinese EFL learners participated in this study, forming three types of proficiency pairing, including 12 high-high pairs, 12 high-low pairs, and seven low-low pairs. All dialogs were audio-recorded and analyzed for dyadic patterns, as well as frequency, types, and solutions of Language-Related Episodes (LREs). The findings indicated that lexis-focused LREs took the largest proportion of LREs, followed by discourse-focused LREs and grammar-focused LREs in all groups. However, compared with proficiency pairing, the efficacy of dyadic interactions has a greater impact on L2 learning. Learners displaying collaborative patterns significantly produced more LREs and correctly solved the majority of the conflicts than those in non-collaborative interactions, while neither the total number nor solutions of LREs reached significance in different proficiency pairing groups. Pedagogical implications on implementing CW in L2 writing classrooms are discussed.

## Introduction

Collaborative Writing (CW), an activity that requires two or more learners to co-author a single text with negotiation and shared responsibility, has received widespread attention recently (Storch and Aldosari, 2012; Storch, 2013; Zhang, 2019). Informed by sociocultural theory (Vygotsky, 1978), which considers learning as a socially mediated process, previous studies have explored CW in three main strands. One is process-oriented, addressing Language-Related Episodes (LREs), interaction patterns, and revision behaviors (Storch, 2001, 2002a,b; Storch and Aldosari, 2012). The second strand, which is product-oriented, has analyzed learners’ perceptions and their writing products in terms of complexity, fluency, and accuracy (Storch, 2005; Wigglesworth and Storch, 2009; Dobao, 2012; Chen and Yu, 2019). The third line of research underscores the factors mediating CW, including task type (Storch, 1998, 1999; Swain and Lapkin, 2001), dyadic patterns (Watanabe and Swain, 2007), and learning proficiency (Leeser, 2004; Cen and Wang, 2021; Deng et al., 2021). To date, patterns of interaction and proficiency pairing have been found to be more conducive to second language (L2) learning than others (Leeser, 2004; Watanabe and Swain, 2007; Storch and Aldosari, 2012), but very few studies have compared these two factors to explore which has a larger impact on learning. To address this issue, this study attempts to investigate the impact of dyadic patterns and proficiency pairing on Chinese EFL learners’ L2 learning in CW.

### Regulated learning in collaboration

Regulated learning, an activity that is intentional, goal-oriented, and metacognitive, requires learners to tactically handle their thoughts, behaviors, and emotions for the goal of tasks (Zimmerman and Schunk, 2011; Wang, 2019). Prior research has demonstrated three stages of regulation, namely planning (set specific goals and plan strategies), monitoring (regulate the plan and process), and evaluation (appraise their performance and reflect it on the next task), which occur when learners accomplish a task individually or collaboratively (Zimmerman, 2000; Järvelä and Hadwin, 2013; Wang, 2019).

Regulation has been a quintessential skill in collaborative learning (Hadwin et al., 2011). Hadwin et al. (2011) classified regulation into three modes: self-, co-, and socially shared regulation. Self-regulated learning (SRL) highlights actions of personal, behavioral factors for constructive learning, in which learners consciously activate, preserve, and modify cognition, affect, and behavior to meet the learning targets (Zimmerman, 2011; Teng and Zhang, 2017). That is, each member of the group needs to regulate his or her behaviors to make contributions to the team, finally leading to successful collaborative learning. Nonetheless, SRL alone is insufficient for effective collaborative learning, which requires co-regulation (group members encourage and support each other’s goals and contributions) and socially shared regulation (students in a group collectively construct shared goals and strategies with negotiation) together (Järvelä and Hadwin, 2013; Qiu and Lee, 2020).

Both individual and group regulation are the basis for effective CW, which means that learners in a group need to manage their own and group behavior, motivation, and emotion to achieve common goals (Järvelä and Hadwin, 2013; Wang, 2019). Meanwhile, learners can scaffold each other to facilitate their regulation during the CW process (Vygotsky, 1978; Donato, 1994; Blau and Shamir-Inbal, 2017). In particular, learners can recognize incongruity between their knowledge and targets, and regulate metacognitive strategies for tasks with shared negotiation (Teng M. F., 2021). Recent studies on CW have shown that computer-based pedagogical tools (CBPT) such as whiteboards and wikis can provide conceptual, metacognitive, and strategic scaffolds, activate students’ participation, and improve their confidence, thus fostering self-regulated learning (Järvelä and Hadwin, 2013; Cho and Lim, 2015). Besides, the motivational regulations recently have been proved to greatly impact the EFL writing (Teng and Zhang, 2017; Teng M.F., 2021). However, little is known about the effect of regulated learning in the face-to-face CW context for EFL learners.

### Proficiency pairing and interaction patterns in CW

One concern for teachers implementing collaborative activities is how to best pair students for the best learning outcome. Language proficiency has been adopted as a main solution by analyzing Language-Related Episodes (LREs), which are defined by Swain and Lapkin (1998) as “any part of a dialog where students talk about the language they are producing, question their language use, or correct themselves or others” (p.326). The frequency, types, and outcomes of LREs have been compared in different proficiency pairing groups, but the results are somewhat mixed. Early studies (Leeser, 2004; Kim and McDonough, 2008; Storch and Aldosari, 2012) have revealed that High-High (H-H) dyads generated a larger number of LREs than High-Low (H-L) and Low-Low (L-L) dyads did, but recent research has suggested the greatest number of LREs was produced by L-L pairs (Niu et al., 2018; Deng et al., 2021). Regarding the types of LREs, some revealed that higher proficiency groups focused on grammatical items and lower proficiency dyads on lexical ones (Leeser, 2004; Storch and Aldosari, 2012), while others reported that learners tended to pay more attention to grammar than lexis regardless of pairs’ proficiency (Kim and McDonough, 2008). Concerning the outcome of LREs, the findings consistently show that H-H pairs could successfully solve more LREs than H-L and L-L groups (Niu et al., 2018; Nguyen and Newton, 2019; Cen and Wang, 2021; Deng et al., 2021; Zabihi and Ghahramanzadeh, 2022). However, these studies, only examining the effect of proficiency pairing, have ignored the efficacy of interaction patterns, another important element that exerts on L2 learning.

One of the representative studies on the nature of interaction is longitudinal investigation of Storch (2002a) in ESL classrooms. She built a model of dyadic interaction based on equality and mutuality, which refer to “the degree of control or authority over the task” and “the level of engagement with each other’ contribution,” respectively (Storch, 2002a, p.127). Four distinct patterns in pairs were identified, including collaborative, expert/novice, dominant/dominant, and dominant/passive. Employing scheme of dyadic interaction of Storch (2002a); Watanabe and Swain (2007) compared the interactions of four core learners co-constructing an essay with four higher- and four lower-proficiency learners, and found patterns of interaction, instead of proficiency pairing greatly impacted frequency of LREs and learners’ performance in post-tests. Similarly, by analyzing pair dialogs produced by 30 EFL learners, Storch and Aldosari (2012) also claimed that dyadic relationships may be more critical than proficiency pairing in L2 learning. However, given the small scale of the research, the results may not be generalizable in other contexts. To the best of our knowledge, only these two studies have compared the effect of dyadic patterns and proficiency pairing in CW, but no statistical tests were performed and neither the solutions of LREs were considered. Thus, it remains unclear to what extent learners’ L2 learning is influenced by these two factors.

To address this issue, this study seeks to investigate the effect of dyadic patterns and proficiency pairing on Chinese EFL learners’ L2 learning in collaborative writing. Two research questions are addressed: (1) How do dyadic patterns and proficiency pairing impact the occurrence of LREs in CW? and (2) How do dyadic patterns and proficiency pairing impact the solutions of LREs in CW?

## Methodology

### Context and participants

This study was conducted in a freshman College English course at a comprehensive university in central China. The course lasts 16 weeks, aiming to facilitate learners’ listening, speaking, reading, and writing skills of English. After being informed of the purpose and nature of the study at the beginning of the second semester, 62 non-English major students (38 males, 24 females) in three classes, aged from 18 to 21, voluntarily participated in this study. They are all taught by the same instructor who has teaching experience of over 20 years. The participants were provided written informed consent and allowed to withdraw from the study at any time. They are all high-intermediate level EFL learners, and have been learning English for 10–11 years. Prior to the study, they had been familiar with pair work in class, but none of them had experience in collaborative writing.

Learners’ L2 proficiency was measured by the score of the English test in the National College Entrance Examination (NMET) and that of the final exam of the College English course in the previous semester, as well as the evaluation of their teacher. The College English course, a compulsory course for the first-year non-English majors, aims to improve students’ overall English proficiency. Specifically, high-proficiency learners were students whose scores were above 130 in NMET or above 80 in the previous semester, and low-proficiency ones were students with scores below 120 in NMET or below 70 in the previous semester. Afterward, the instructor helped to assess students’ proficiency level when we encountered difficulty. Finally, 36 high-proficiency and 26 low-proficiency students were identified.

To verify our identification of participants’ L2 proficiency, one argumentative writing task was individually implemented as a pre-test and rated by two raters based on scoring rubrics of CET 4 (intercoder reliability *r* = 0.93). Argumentative writing, the most popular and standardized assessment for L2 learners, is effective to evaluate learners’ academic achievements in terms of linguistic competence and critical thinking (Teng and Zhang, 2017, 2020). Results of independent samples *t*-test implicated a significant difference in the writing scores (*t* = 3.14, *p* < 0.05), suggesting heterogeneous proficiency between high-proficiency learners (M = 12.80, SD = 0.92) and low-proficiency counterparts (M = 11.96, SD = 1.16). Guided by the study of Leeser (2004), 62 participants were paired into three groups, including 12 high-high (H-H), 12 high-low (H-L), and seven low-low (L-L) dyads.

### Procedures

The study lasted 8 weeks and multiple sources of data, including questionnaire, writing essays, screen recording, and audio recording, were collected. In the first 3 weeks, an open-ended questionnaire was distributed to all students to obtain basic information, such as their ages, majors, English scores of NMET and College English course, as well as their interest in CW. Sixty-two students reported in the questionnaire that they were willing to take part in the study and their proficiency was evaluated by the grades of NMET and English course, as well as the instructor’s assessment.

In week 4, participants were required to individually write a College English Test Band 4 (CET 4) essay in class, the topic of which is “Listening is more important than talking.” They were required to write an argumentative essay of 120–180 words within 30 min. In week 5, participants were informed of the group assignment and the pre-writing preparation (including the guidance of using EV capture on a computer).

The collaborative task was conducted in week 6. First, the instructor provided the video-based pre-task modeling along with the suggestions on collaborative writing to prepare the students for the writing task. Subsequently, each pair, using one computer, was asked to finish a CET 4 writing task on the topic “Take a job or go to a graduate school” within 45 min. As dyads take longer to complete tasks than individuals (Storch, 1999, 2005), a longer time was provided for pairs. During the process, all discussions and dialogs were recorded by EV capture and smartphones.

### Data analysis

After data collection, 31 dialogs were transcribed and operationalized as LREs, which indicate the language learning in collaborative writing. Following Niu et al. (2018), LREs were categorized based on what aspect of language learners tackled: whether lexical (L-LREs), grammatical (F-LREs), or discursive (D-LREs). L-LREs focused on word choice, word meaning, word use, and spelling; F-LREs dealt with grammatical issues such as tense, preposition, article, and S-V agreement, and D-LREs with sentence patterns and sentence link devices.

We also coded LREs in terms of the nature of the solution, identifying LREs that are successfully resolved (√) or unresolved (?). Successfully resolved LREs are those in which linguistic issues are solved correctly either *via* self-correction or other correction. Unresolved LREs are defined as those in which group members cannot solve the problem based on their current knowledge or work out an incorrect result. The following are some examples:

Excerpt 1, from the data of a H-L dyads, provides an example for L-LREs. As L22 suggested using “nowadays” to replace “today,” H32 adopted it and this word choice problem was successfully solved.

Excerpt 1 H32L22/L-LREs/Word choice/Successfully resolved.

1. *H32: Today, there are some people…*
2. *L22: We can use nowadays*.
3. *H32: What?*
4. *L22: The word “nowadays” seems to be more advanced than “today.”*
5. *H32: That makes sense*.

Excerpt 2 comes from another H-L pair who are discussing the forms of “talent.” Without too much negotiation, the incorrect form “talent” was used.

Excerpt 2 H28L18/F-LREs/Single-plural/Unresolved.

1. *H28: For contemporary talent*.
2. *L18: Should we use the plural form of talent?*
3. *H28: Should we? Maybe single form is right*.
4. *L18: Ok*.

Excerpt 3 shows an example of D-LREs. L5 and L6 were constructing a sentence that was beyond their capacity, and this linguistic issue was unresolved as L6 kept silent.

Excerpt 3 L5L6/D-LREs/Sentence pattern/Unresolved.

1. *L5: Sui zhe shi dai de fa zhan* (*Chinese for “with the development of the times”*).
2. *L6: With the development…*
3. *L5: We have used “with” here, do you have any other expression?*
4. *L6: ……*

Guided by framework of Storch (2002a), the dyadic patterns were identified as collaborative, expert/novice, dominant/dominant, and dominant/passive, which are different in the level of equality and mutuality. Specifically, learners who exhibit collaborative interactions frequently negotiate, provide feedback, request, and explanation, and support each other. In an expert/novice pattern, one participant is more likely to control the task, but he/she endeavors to encourage the novice to make contributions. In contrast, dominant/dominant patterns are defined as those in which disagreements frequently exist regardless of the equal contribution to the task. Dominant/passive interactions are those in which one learner controls the task with self-directed requests and questions, while the other contributes little. Discussion and peer assistance rarely take place in these interactions.

The following excerpts demonstrate four patterns in the data. Excerpt 4 comes from a H-H pair, who were discussing word choice with negotiation, request, and repetition, showing a collaborative pattern.

Excerpt 4 Collaborative-H9H10.

1. *H10: Everyone has different choices*.
2. *H9: “Their choices are diverse,” we can use this word “diverse.”*
3. *H10: Diverse? Which word?*
4. *H9: D-I-V-E-R-S-E*.
5. *H10: Oh, diverse, the choices are diverse*.
6. *H9: There is another word that refers to diversity*.
7. *H10: Diversity means “duoyangxing.”*
8. *H9: I mean an adjective, ending with o-u-s*. *Oh, V-A-R-I-O-U-S*.
9. *H10: See this word, multifarious, which is advanced and has more explanations, and I am afraid that the teacher cannot recognize it*.
10. *H9: Hahahaha, ok*.

Excerpt 5 illustrates the pattern of expert/novice. H30 played the role of “expert” and invited L20 to interact with her.

Excerpt 5 Expert/novice-H30L20.

1. *H30: How to spell “below.”*
2. *L20: B-E-L-O-W*.
3. *H30: B-E-L-O-W, our or ours?*
4. *L20: We use “our” directly*.
5. *H30: Oh, yes*. *I want to write “the person who has a higher degree of education will more likely to be admitted.” What do you think of it?*
6. *L20: I think this sentence is great*.
7. *H30: Ok*.

Excerpt 6 shows the dominant/dominant interaction. Although both H35 and L25 made contributions, they were unwilling to negotiate and reach agreement.

Excerpt 6 Dominant/dominant-H35L25.

1. *H35: What Do You want To write next?*
2. *L25: The purpose of working is for money and financial independence, but the success in the future is different if you continue studying*.
3. *H35: Oh my god*.
4. *L25: What?*
5. *H35: You are away from the topic*.
6. *L25: Why?*
7. *H35: The passage just asks you to choose between working and going to a graduate school, but you say…It’s ok to write about financial independence, while the interest you say is surplus*.
8. *L25: Why is it surplus? We need the transition*.
9. *H35: How does going to a graduate school increase the interest?*
10. *L25: No, I mean academic research*.
11. *H35: Is it related to daily life?*
12. *L25: It must be*.
13. *H35: How does going to a graduate school relate to the daily life?*
14. *L25: It must have correlations*.
15. *H35: Do not say it anymore*.

Excerpt 7, a part of the dialog of two low-proficiency learners, provides an example of a dominant/passive interaction. As the excerpt shows, L8 often produced long monologues and requests, but L7 was passive and reluctant to contribute.

Expert 7 Dominant/passive-L7L8.

1. *L8: How do we change our topic to academic research?*
2. *L7: emmmm*.
3. *L8: ‘For some people’, which connectives should we use? ‘We feel more pressure from our peers through constant competition, demanding us to go to graduate school.’ Now we have already written 114 words and we can write it in 120 words to satisfy requirements*.
4. *L7: yes*.
5. *L8: Let us make an end! How to make it? We must say something related to success*.
6. *L7: Ok*.
7. *L8: How to say it? How to say “zhixiang?” The meaning of destiny is various, the road leading… leading? Can we use ‘leading’?*
8. *L7: Yes*.

For inter-rater reliability, 10% of the data were independently coded by two raters, achieving an agreement of 90, 92, and 91% for linguistic focus, solutions, and dyadic patterns, respectively. All disagreements were resolved with discussion. The remaining data were coded twice by the second researcher with 2 weeks’ interval. We counted the frequency of occurrences and percentage of solutions for LREs, and examined the effect of proficiency and dyadic patterns on language learning using one-way ANOVA. The significance level was set at 0.05.

## Results

### Occurrence of LREs

A total of 1,019 LREs and four dyadic patterns were identified in 31 pairs’ dialogs. Table 1 presents the number of L-LREs, F-LREs, and D-LREs in different dyadic interactions. Learners who formed collaborative (M = 38.29) and expert/novice patterns (M = 37.50) tended to generate more LREs than dominant/dominant (M = 21.50) and dominant/passive ones (M = 22.00), but all groups focused on the L-LREs (collaborative: 63.59%, expert/novice: 64.00%, dominant/dominant: 58.14%, and dominant/passive: 62.88%). To examine the significance of this trend, a series of one-way ANOVAs was performed. Dyadic pattern was employed as an independent variable, and the frequency of LREs and linguistic focus as dependent variables. The assumptions of normality and homogeneity of variances, checked by the Kolmogorov–Smirnov test (*p* > 0.05) and Levene test (*p* > 0.05), were met for the data.

**Table 1:** The frequency and type of Language-Related Episodes (LREs) by dyadic patterns.

Types of LREs	/	Collaborative (*n* = 17)	Expert/ novice (*n* = 4)	Dominant/dominant (*n* = 4)	Dominant/passive (*n* = 6)	Total
L-LREs	N/%	414 (63.59%)	96 (64.00%)	50 (58.14%)	83 (62.88%)	643
	M	24.35	24.00	12.50	13.83	/
	SD	6.40	10.13	3.00	4.17	/
F-LREs	N/%	98 (15.05%)	27 (18.00%)	10 (11.63%)	20 (15.15%)	155
	M	5.76	6.75	2.50	3.33	/
	SD	2.71	4.43	1.92	1.51	/
D-LREs	N/%	139 (21.35%)	27 (18.00%)	26 (30.23%)	29 (21.97%)	221
	M	8.18	6.75	6.50	4.83	/
	SD	4.04	3.86	3.11	1.60	/
Total	N/%	651 (100%)	150 (100%)	86 (100%)	132 (100%)	1,019
	M	38.29	37.50	21.50	22.00	32.87

Results of the one-way ANOVA show a significant effect of dyadic patterns [*F* (3, 27) = 7.321, *p* < 0.05], *post hoc* test indicates that learners in collaborative interaction significantly discussed more linguistic issues than those in dominant/dominant (*p* < 0.05) and dominant/passive patterns (*p* < 0.05). The same trend was obtained with the distribution of L-LREs [*F* (3, 27) = 6.866, *p* < 0.05] across four conditions, while no significant difference among groups is observed regarding both F-LREs [*F* (3, 27) = 2.901, *p* > 0.05] and D-LREs [*F* (3, 27) = 1.360, *p* > 0.05].

Concerning the occurrence of LREs producing in different proficiency groups, the results are shown in Table 2. On average, the H-L pairs generated a greater number of LREs (M = 36.33) than the H-H (M = 33.25) and L-L pairs (M = 26.29) did. As for the types of LREs, three groups generated a similar percentage of L-LREs (H-H: 62.91%, H-L: 63.30%; and L-L: 63.04%), followed by D-LREs and F-LREs. However, no significant difference was found neither in the distribution of total number of LREs [*F* (2, 28) = 1.751, *p* > 0.05] nor its subcategories [L-LREs: *F* (2, 28) = 1.495, *p* > 0.05; F-LREs: *F* (2, 28) = 3.755, *p* > 0.05; D-LREs: *F* (2, 28) = 0.011, *p* > 0.05] among three groups.

**Table 2:** The frequency and type of LREs by proficiency pairing.

Types		H-H (*n* = 12)	H-L (*n* = 12)	L-L (*n* = 7)	Total
L-LREs	N/%	251 (62.91%)	276 (63.30%)	116 (63.04%)	643
	M	20.92	23.00	16.57	/
	SD	7.62	8.63	6.55	/
F-LREs	N/%	61 (15.29%)	75 (17.20%)	19 (10.33%)	155
	M	5.08	6.25	2.71	/
	SD	2.58	3.31	1.50	/
D-LREs	N/%	87 (21.80%)	85 (19.50%)	49 (26.63%)	221
	M	7.25	7.08	7.00	/
	SD	3.96	3.29	4.24	/
Total	N/%	399 (100%)	436 (100%)	184 (100%)	1,019
	M	33.25	36.33	26.29	32.87

### Solutions of LREs

Concerning whether dyadic pattern affects the outcomes of LREs, the results are shown in Table 3. Generally, each pair successfully resolved most LREs. Learners in collaborative (85.71%) and expert/novice patterns (85.33%) were able to solve more LREs correctly than those in dominant/dominant (77.91%) and dominant/passive patterns (75.00%). The percentage of solved LREs out of the total LREs in each group was analyzed through one-way ANOVA with *post-hoc* analysis. The results indicated a significant effect of dyadic patterns [*F* (3, 27) = 4.438, *p* < 0.05], with learners in collaborative patterns significantly and correctly solving more language problems than those in dominant/passive ones (*p* < 0.05). Regarding the unresolved LREs, there was also a significant difference in groups of different dyadic patterns [*F* (3, 27) = 4.438, *p* < 0.05], with more language problems abandoned by learners in dominant/passive interactions than those in collaborative ones (*p* < 0.05).

**Table 3:** Solutions of LREs by dyadic patterns.

Solutions	/	Collaborative (*n* = 17)	Expert/novice (*n* = 4)	Dominant/dominant (*n* = 4)	Dominant/ passive (*n* = 6)	Total
Resolved	N/%	558 (85.71%)	128 (85.33%)	67 (77.91%)	99 (75.00%)	852
	M	32.82	32.00	16.75	16.50	/
	SD	6.21	15.56	6.40	6.66	/
Unresolved	N/%	93 (14.29%)	22 (14.67%)	19 (22.09%)	33 (25.00%)	167
	M	5.47	5.50	4.75	5.50	/
	SD	3.06	3.11	0.96	2.26	/
Total	N/%	651 (100%)	150 (100%)	86 (100%)	132 (100%)	1,019

Table 4 is the solutions resolved by different proficiency groups. Similarly, learners in three groups could successfully resolve most language issues. The H-H and H-L pairs tackled more linguistic issues than the L-L dyads did. However, results of one-way ANOVA show that there were no significant differences in neither successfully resolved [*F* (2, 28) = 3.195, *p* > 0.05] nor unresolved LREs [*F* (2, 28) = 3.195, *p* > 0.05], implying that proficiency pairing had little impact on the outcomes of LREs.

**Table 4:** Solutions of LREs by proficiency pairing.

Solutions	/	H-H (*n* = 12)	H-L (*n* = 12)	L-L (*n* = 7)	Total
Resolved	N/%	340 (85.21%)	372 (85.32%)	140 (76.09%)	852
	M	28.33	31.00	20.00	/
	SD	9.40	11.46	8.87	/
Unresolved	N/%	59 (14.79%)	64 (14.68%)	44 (23.91%)	167
	M	4.92	5.33	6.29	/
	SD	2.23	2.54	3.55	/
Total	N/%	399 (100%)	436 (100%)	184(100%)	1,019

## Discussion and implications

This study investigated whether dyadic patterns or proficiency pairing influences Chinese EFL learners’ L2 learning. In general, the former had a greater impact on the occurrences and solutions of LREs. In addition, all groups tended to focus more on lexical issues when discussing linguistic issues.

Concerning the frequency of LREs, learners who engaged in collaborative patterns significantly generated a greater number of LREs than those in non-collaborative (dominant/dominant, dominant/passive) interactions, but there was no significant difference in groups of different proficiency pairing. The findings were in line with those of Watanabe and Swain (2007), but stood in opposition to the argument that proficiency pairing was a critical factor mediating the frequency of LREs (Leeser, 2004; Deng et al., 2021). This could suggest that learners benefited more from collaborative interactions, where both group members were actively involved in the co-construction of knowledge and negotiated problems through explanations, requests, and repetitions (Storch, 2002a; Watanabe and Swain, 2007). Thus, language can be used as a tool to facilitate language acquisition. However, proficiency differences did not necessarily influence the nature of language learning, which can be corroborated by the mixed findings of previous research that the greatest quantity of LREs may be produced either by H-H or L-L dyads (Leeser, 2004; Deng et al., 2021).

Regarding the focus of LREs, each group, regardless of the dyadic patterns and proficiency, produced the largest proportion of lexical items. This is in line with the results of numerous previous studies (Wigglesworth and Storch, 2009; Storch and Aldosari, 2012; Niu et al., 2018; Deng et al., 2021). Two factors can explain the findings. One is the task type. Argumentative writing tends to elicit more lexical LREs compared with grammar-based tasks such as dictogloss and text reconstruction (Swain and Lapkin, 1998; Storch, 1999). In addition, compared with more structured tasks (focus on the grammar, e.g., dictogloss), the writing task is less structured where learners focus on the content (Storch, 1998). Thus, learners’ attention was drawn to lexis, including word choice, word meaning, and spelling. Another factor is the L2 proficiency level of the participants. They were high-intermediate learners and their needs for grammatical accuracy may not be as strong as lower-proficiency learners recruited in other studies (Wigglesworth and Storch, 2009). Therefore, more lexical items were discussed in the process.

Consistent with previous findings (Niu et al., 2018; Cen and Wang, 2021), this study found that all groups successfully resolved a large proportion of LREs. Although there was no significant difference in proficiency pairing, the H-H and H-L pairs were able to solve more LREs correctly than the L-L pairs. Due to the limited L2 knowledge, the low proficiency learners encountered difficulties working out linguistic issues, leading to the high proportion of LREs unresolved (Storch, 2013). Leeser (2004) argued that low-proficiency learners had more opportunities to discuss and learn language only when they are paired with a higher-proficiency counterpart, while a high-proficiency one could benefit more with a similar high-proficiency learner. Nevertheless, our study implicated that both proficiency pairing and dyadic patterns should be considered to discuss this issue.

Learners with collaborative orientation significantly solved more LREs than those with dominant/passive pattern, suggesting that pairing different proficiency learners is helpful to promote L2 learning only when they are collaborative (Watanabe and Swain, 2007). Scaffolding is more likely to occur among pairs in collaborative patterns, with both group members affording to the resolution of LREs (Donato, 1994). That is, learners actively pool their linguistic resources to co-construct the knowledge and work out linguistic problems together, which gives them more opportunities to interact with each other and facilitate language learning (Donato, 1994; Dobao, 2012). Moreover, from the transcriptions of dialogs, learners in collaborative patterns were found to frequently deploy motivational (emotional control), cognitive (e.g., course memory), metacognitive (e.g., idea planning), and social behavior strategies (e.g., feedback handling and peer learning), which illustrated multi-dimensional structure of SRL (Teng and Zhang, 2017; Teng L.S., 2021). For example, with peer feedback, learners actively used the word they have learned in class to successfully solve the language problems, which suggests that regulation is critical to CW for raising learners’ awareness to control and enhance the writing process, build knowledge construction, and promote language learning (Qiu and Lee, 2020; Teng and Zhang, 2020). In addition, learners’ cognitive load in utilizing linguistic knowledge for writing essays can be greatly relieved by active peer interactions and discussions (Teng L.S., 2021). With common goals, each member in group co-regulated this collective task through feedback and negotiation, understanding what peers are thinking and making adaptations, thus supporting early activities of planning so that they could finally evaluate the process and outcome (Järvelä and Hadwin, 2013; Teng M.F., 2021).

Overall, the findings have pedagogical implications on how to implement collaborative writing in EFL classrooms. Students should be encouraged to regulate the collaborative process so as to foster L2 learning. For example, teachers could guide students with examples about how to use different SRL strategies in cognition, metacognition, and social behavior to promote writing process (Teng and Zhang, 2020). Besides, our findings suggest that learners in different proficiency groups gained knowledge only when they displayed collaborative interaction. Thus, it is essential to consider dyadic patterns and how they are formed (Watanabe and Swain, 2007). Firstly, provided with the pre-task modeling such as video clip and teachers’ instruction that encourage collaborative pattern, students could discuss the merits of collaborative writing and how to handle disagreements before the task. Secondly, after collaborative writing, teachers could invite students who gained from peer assistance to share their experience in the process. This can help students hold a positive attitude toward CW and learn how to effectively collaborate with their partners, fostering collaborative interaction in the next activity.

Some limitations of the study should be acknowledged. Firstly, this study employed a one-shot design and cannot observe learners’ L2 development over time. Future studies could explore the longer-term impact of dyadic patterns and proficiency pairing on L2 learning by conducting multiple CW tasks. Secondly, learners’ proficiency was assessed by combining scores of NMET, English course, and teachers’ evaluation, which may not be accurate. Thus, a more valid and standardized measure can be used in future research.

## Data availability statement

The raw data supporting the conclusions of this article will be made available by the authors, without undue reservation.

## Ethics statement

The studies involving human participants were reviewed and approved by the Wuhan University of Technology. The patients/participants provided their written informed consent to participate in this study.

## Author contributions

NL and YL conceived of the initial idea, designed the study, revised subsequent versions and proofread the manuscript. NL collected the data, analyzed the data, and drafted the manuscript. YL finalized the manuscript for submission as the corresponding author. All authors contributed to the article and approved the submitted version.

## Funding

This study was funded by China Social Science Research Foundation, Project Number: 19BYY229.

## Conflict of interest

The authors declare that the research was conducted in the absence of any commercial or financial relationships that could be construed as a potential conflict of interest.

## Publisher’s note

All claims expressed in this article are solely those of the authors and do not necessarily represent those of their affiliated organizations, or those of the publisher, the editors and the reviewers. Any product that may be evaluated in this article, or claim that may be made by its manufacturer, is not guaranteed or endorsed by the publisher.
